# The efficacy and safety of the infiltration of the interspace between the popliteal artery and the capsule of the knee block in total knee arthroplasty: A prospective randomized trial protocol

**DOI:** 10.1097/MD.0000000000021670

**Published:** 2020-08-14

**Authors:** Zhongxiao Cong, Lejun Zhang, Fengying Ma

**Affiliations:** Department of Operating Room, Qilu Hospital of Shandong University, Shandong, China.

**Keywords:** adductor canal block, interspace between the popliteal artery and capsule of the knee, pain, prospective, protocol, total knee arthroplasty

## Abstract

**Background:**

Total knee arthroplasty (TKA) is an established and successful surgical procedure which is the major treatment for degenerative knee joint diseases. A novel technique to address posterior knee joint pain is the infiltration of local anesthetic between the interspace between the popliteal artery and capsule of the knee (IPACK). The goal of this randomized clinical trial was to assess the efficacy and safety of adding IPACK to adductor canal block (ACB) after TKA.

**Methods:**

This was a prospectively randomized trial that investigated the effectiveness and safety of the IPACK after TKA. Approval from Clinical Studies Ethical Committee in Qilu Hospital of Shandong University was obtained. The inclusion criteria were adult patients undergoing primary unilateral TKA and American Society of Anesthesiologists grade 1 or 2 with normal cognitive function. The patients were randomized to 1 of 2 treatment options: ACB-alone group and ACB + IPACK group. The primary outcome was the total morphine consumption during postoperative 24 hours. Secondary outcomes included postoperative pain score, time to first and total dosage of rescue morphine in postoperative 48 hours, early and late postoperative period (from postoperative day 0–3 months follow-up) performance-based test (Timed-Up and Go test, and quadriceps strength). Postoperative nausea and vomiting, length of hospital stay, patient satisfaction, and other adverse events were also evaluated.

**Results:**

It was hypothesized that when combined with a control group, the IPACK block would result in a lower morphine consumption and pain score after TKA.

**Trial registration:**

This study protocol was registered in Research Registry (researchregistry5765).

## 1 Introduction

Total knee arthroplasty (TKA) is an established and successful surgical procedure which is the major treatment for degenerative knee joint diseases. With the popularity and promotion of TKA, increasing numbers of patients with degenerative knee diseases are undergoing this surgery to restore knee function and mobility, as well as to improve quality of life.^[1–3]^ However, severe pain after TKA makes it difficult for many patients to participate in early postoperative rehabilitation and to exercise, which might result in subsequent unsatisfactory recovery of knee joint function and great reduction in patients’ quality of life. Perioperative pain control has direct influence on postoperative recovery and surgical outcome. An appropriate perioperative analgesic protocol could relieve postoperative pain and allow functional exercising, leading to early rehabilitation.^[4]^

Femoral nerve block (FNB) is one of the most commonly used pain-relief methods, which has been proven to be effective on relieving the pain, reducing the usage of opioid painkiller, and shortening the hospital stays. Moreover, FNB is regard as the gold standard for postoperative analgesia after TKA by some surgeons. However, FNB may lead to postoperative quadriceps weakness, which not only limits the patients’ ambulation and early physical rehabilitation, but also increases the risk of falling. These deficiencies make the rehabilitation results unsatisfactory.^[5–8]^ Recently, adductor canal block (ACB) has been demonstrated to be an effective alternative to the femoral nerve block, providing similar analgesic efficacy while sparing the motor strength significantly. Both ACB and FNB provide analgesia primarily to the anterior medial part of the knee, and as a result, TKA patients typically require supplemental multimodal analgesia, including opiates, to address posterior joint pain.^[2,9]^

A novel technique to address posterior knee joint pain is the infiltration of local anesthetic between the interspace between the popliteal artery and capsule of the knee (IPACK). IPACK provides analgesia to the posterior compartment of the knee without compromising foot strength.^[10]^ Indeed, a cadaveric study demonstrated that an IPACK injection surrounded the middle genicular artery with injectate, supporting the hypothesis that local anesthetic infiltration surrounding the articular sensory nerves near the middle genicular artery is a potential mechanism of analgesia for the IPACK. This study also showed potential for the injectate to contact the tibial and commonperoneal nerves, so an IPACK block could potentially result in sensory and motor deficits below the knee.^[11]^

However, studies evaluating the effect of IPACK block on postoperative knee pain after TKA are lacking.^[12–14]^ As such, the goal of this randomized clinical trial was to assess the efficacy and safety of adding IPACK to ACB after TKA. It was hypothesized that when combined with a control group, the IPACK block would result in a lower morphine consumption and pain score after TKA.

## 2 Materials and methods

### 2.1 Study design

**Figure 1:**
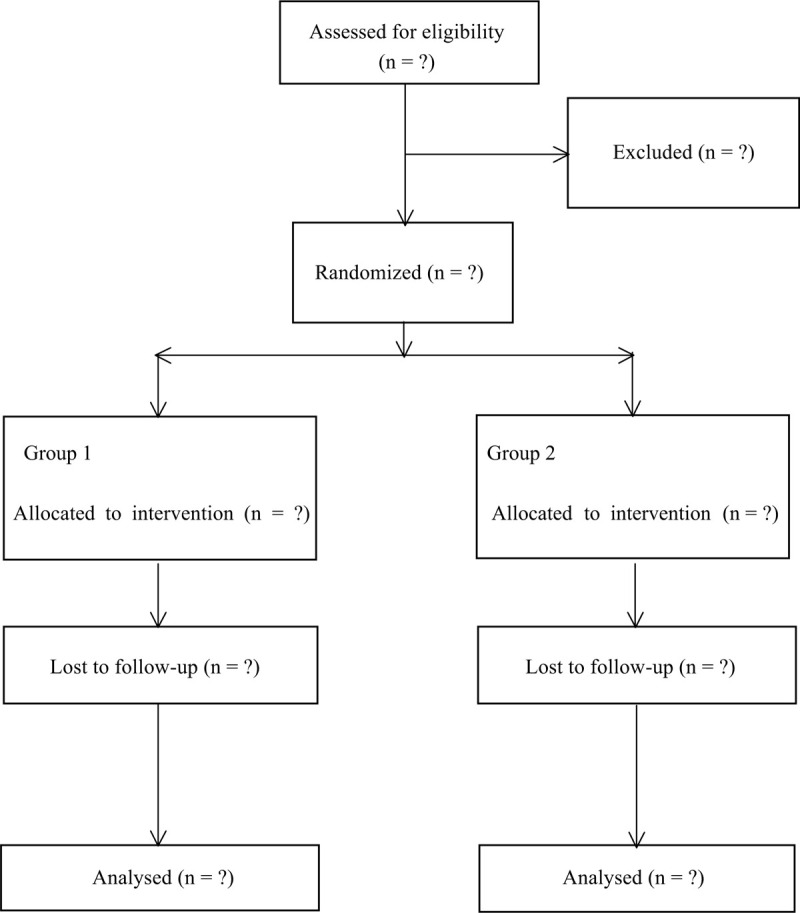
Flow diagram of the study.

This was a prospectively randomized trial that investigated the effectiveness and safety of the IPACK after TKA. Approval from Clinical Studies Ethical Committee in Qilu Hospital of Shandong University was obtained (DSA793002). This study has been published at the Research Registry (researchregistry5765). We followed the Consolidated Standards of Reporting Trials (CONSORT) guidelines for reporting randomized trials and provided a CONSORT flow diagram (Fig. 1).

### 2.2 Patients

The inclusion criteria were adult patients undergoing primary unilateral TKA and American Society of Anesthesiologists grade 1 or 2 with normal cognitive function. The exclusion criteria were the following: patients unwilling to participate, poorly controlled diabetes, history of inflammatory arthritis, nonambulatory/bed ridden patients, known allergy to the anesthetic drugs, history of bleeding disorder, history of arrhythmia or seizures, sepsis, and pre-existing lower extremity neurological abnormality.

### 2.3 Randomization and blinding

The patients were randomized to 1 of 2 treatment options: ACB-alone group and ACB + IPACK group. Randomization was performed without any stratification. Randomization listings were prepared with a probability of 0.4 to 0.6 and after that, randomization letters were printed according to the results of the randomization. After the patient had given consent, a member of the in-hospital clinical study center chose 1 of the 2 letters and the patient was assigned to 1 group. Patients, surgeons, anesthesiologists, nurses, and research assistants collecting data were blinded to group allocation.

### 2.4 Interventions

The patients were admitted to the operating room without any premedication. Spinal anesthesia was administered by means of a 22-gauge spinal needle, consisting of 15 mg (3 mL) of 0.5% spinal bupivacaine to each patient after both ACB and ACB + IPACK. All operations were performed by the same surgical team using a similar technique.

For patients in the ACB-alone and ACB + IPACK groups, an ACB was performed in the preoperative block area by the regional anesthesia team prior to surgery. This team included an attending anesthesiologist who performed or oversaw the ACB. The ACB was performed under ultrasound guidance at the midlevel of the thigh (at the midpoint between the anterior superior iliac spine and the superior pole of the patella) using 15 mL of 0.5% bupivacaine. For patients in the ACB + IPACK group, IPACK blocks were performed under ultrasound guidance with a 5 to 2 MHz curvilinear transducer using a 22 G 4-in. Chiba-type spinal needle. The anesthesiologist identified the popliteal artery, in a short-axis view, at the popliteal crease and moved cephalad just beyond the femoral condyles, at the level where the condyles merge with the shaft of the femur. The tibial and peroneal nerves were visualized superficial to the popliteal artery. After identifying the space between the femur and popliteal artery, the needle was advanced in-plane from medial to lateral. The tip was positioned at the middle of the femur and near the lateral border near the periosteum. Subsequently, 5 to 10 mL of local anesthetic was injected to ensure adequate spread to the lateral end of the femur. On withdrawing the needle, the anesthesiologist further injected the rest of the 25 mL of 0.25% bupivacaine along the femur, infiltrating 5 mL incrementally in the area between the artery and femur, and finishing at the medial end of the femur.

### 2.5 Postoperative protocol

All subjects underwent a standard preoperative and postoperative multimodal pain management regimen. Preoperative medications, which included acetaminophen, oxycodone, celecoxib, and gabapentin, were given in the preoperative area 1 hour prior to surgery. Postoperative medications included acetaminophen, ketorolac followed by celecoxib (for 3 months), gabapentin (standing order for 10 days), oral opioids (as needed), and intravenous hydromorphone for breakthrough pain.

### 2.6 Outcome measures

The primary outcome was the total morphine consumption during postoperative 24 hours. Secondary outcomes included postoperative pain score, time to first and total dosage of rescue morphine in postoperative 48 hours, early and late postoperative period (from postoperative day 0–3 months follow-up) performance-based test (Timed-Up and Go test, and quadriceps strength). Postoperative nausea and vomiting, length of hospital stay, patient satisfaction, and other adverse events were also evaluated.

### 2.7 Sample size calculation

The sample size calculation was based on a pilot study that we conducted on 16 patients (whose data were not included in the present study). In this prior study, the mean difference and standard deviation of the pain scores 24 hours after the operation between the ACB and ACB + IPACK groups were 0.40 and 0.19, respectively. From this, it was determined that 39 subjects would be required to reach an *α* value of 0.05 and a power of 85%. It was estimated that the attrition rate due to canceled surgery or reasons of late patient ineligibility could be up to 20% and, therefore, to account for this, the final sample size selected was n = 94 (47 per group).

### 2.8 Statistical analysis

Data were analyzed using the statistical software package SPSS version 25.0 (Chicago, IL). Continuous variables were described as the mean ± standard deviation, and differences between groups were analyzed using a series of one-way analysis of variance (ANOVA) with Bonferroni post-hoc test, while differences between groups over time were analyzed using multi-way ANOVA with Bonferroni post-hoc test. Categorical variables were described as the number (%), and were analyzed by Fisher exact test. A *P* value of <.05 was considered statistically significant.

## 3 Discussion

As clinicians improve healthcare, a common goal is to maintain quality measures while reducing hospital length of stay. Advancements in postoperative regional analgesia, specifically the introduction of the ACB, have been pivotal in optimizing care for patients undergoing TKA. Although ACB is effective, it primarily addresses anterior knee pain, and patients often need supplemental analgesia to address posterior knee pain. The posterior knee capsule is innervated by the terminal branches, which ramify from the popliteal plexus mainly formed by the contribution of the tibial and posterior branch of the obturator nerves. A novel technique to address posterior knee joint pain is the infiltration of local anesthetic between the IPACK. IPACK provides analgesia to the posterior compartment of the knee without compromising foot strength. The goal of this randomized clinical trial was to assess the efficacy and safety of IPACK after TKA.

## Author contributions

**Conceptualization:** Fengying Ma.

**Data curation:** Lejun Zhang.

**Formal analysis:** Lejun Zhang.

**Funding acquisition:** Zhongxiao Cong.

**Investigation:** Zhongxiao Cong, Lejun Zhang.

**Methodology:** Zhongxiao Cong.

**Resources:** Zhongxiao Cong.

**Software:** Lejun Zhang.

**Supervision:** Fengying Ma.

**Validation:** Fengying Ma.

**Visualization:** Fengying Ma.

**Writing – original draft:** Zhongxiao Cong, Lejun Zhang.

**Writing – review & editing:** Fengying Ma.
